# Effects of Long Chain Omega-3 Polyunsaturated Fatty Acids on Brain Function in Mildly Hypertensive Older Adults

**DOI:** 10.3390/nu10101413

**Published:** 2018-10-02

**Authors:** Peter R. C. Howe, Hamish M. Evans, Julia C. Kuszewski, Rachel H. X. Wong

**Affiliations:** 1 School of Biomedical Sciences and Pharmacy, Clinical Nutrition Research Centre, University of Newcastle, Callaghan, NSW 2308, Australia; hamish.evans@newcastle.edu.au (H.M.E.); Julia.kuszewski@uon.edu.au (J.C.K.); rachel.wong@newcastle.edu.au (R.H.X.W.); 2 Institute for Resilient Regions, University of Southern Queensland, Springfield Central, QLD 4300, Australia

**Keywords:** cerebrovascular function, cognition, mood, cerebral blood flow, omega-3, hypertension

## Abstract

Purported benefits of long chain omega-3 polyunsaturated fatty acid (LCn-3PUFA) for brain function may be attributable, at least in part, to improved cerebral perfusion. A pilot randomised controlled trial was undertaken to investigate effects of taking a DHA-rich fish oil supplement for 20 weeks on cerebrovascular function, mood and cognitive performance. Borderline hypertensives aged 40–85 years with low habitual LCn-3PUFA intake took four capsules/day of EPAX (1600 mg DHA + 400 mg EPA) or placebo (corn oil). Cerebrovascular function was assessed at baseline and after 20 weeks in 38 completers (19 on each supplement) using transcranial Doppler ultrasound of blood flow in the middle cerebral artery at rest and whilst performing a battery of cognitive tasks (neurovascular coupling). The primary outcome, cerebrovascular responsiveness (CVR) to hypercapnia, increased 26% (*p* = 0.024) in women; there was no change in men. In contrast, neurovascular coupling increased significantly (*p* = 0.01 for the overall response) in men only; the latter correlated with an increase of EPA in erythrocytes (*r* = 0.616, *p* = 0.002). There was no associated improvement of mood or cognition in either men or women. These preliminary observations indicate that LCn-3PUFA supplementation has the potential to enhance blood flow in the brain in response to both hypercapnic and cognitive stimuli. Future studies should examine differential effects of EPA and DHA and take account of the gender differences in responsiveness to supplementation.

## 1. Introduction

Omega-3 (n-3) and omega-6 (n-6) polyunsaturated fatty acids (PUFA) are essential nutrients with a multiplicity of similar and often complementary physiological roles mediated by their functions as structural components of membranes, substrates for eicosanoids and modulators of gene transcription, to name a few. An ever expanding range of potential health benefits has been ascribed to long chain (LC) n-3PUFA, viz. eicosapentaenoic acid (EPA) and docosahexaenoic acid (DHA), as distinct from the shorter chain length n-3PUFA, α-linolenic acid. Whilst structural roles of both n-3 and n-6 PUFA are vital in early development, particularly of the nervous system, their regulatory functions are of critical importance in maintaining physiological homeostasis and counteracting chronic non-communicable, e.g., inflammatory, conditions that impact aging populations. Among these, effects on brain function are emerging as possibly the most important for an aging population. Our early research demonstrated significant benefits of LCn-3PUFA supplementation, particularly with a DHA-rich oil, for both mood and cognition, in older adults with mild cognitive impairment [1]. We hypothesised that such benefits might be attributable, at least in part, to improvement of cerebral blood flow following LCn-3PUFA supplementation, as it seemed plausible that the relatively rapid changes in brain function observed in adults following supplementation were more likely to be mediated by effects on the microvascular endothelium than on neuronal structure and function [2,3]. This hypothesis enabled us to predict that individuals with circulatory impairments, e.g., hypertensives, diabetics and even the elderly and/or obese, might be at heightened risk of mood or cognitive deficits and more likely to respond to supplementation with LCn-3PUFA [3] or similar vasoactive nutrients [4,5,6]. Indeed, we have since explored this hypothesis in adults with type 2 diabetes [7] and in postmenopausal women [8] using transcranial Doppler ultrasound (TCD) as a non-invasive surrogate measure of cerebrovascular function and found inverse correlations between cognitive performance and markers of cerebrovascular compliance and vasodilator responsiveness in these populations. Moreover, we have shown that supplementation with a range of vasoactive supplements or foods including resveratrol [9], wild green oat extract [10] and peanuts [11] have the potential to improve both cerebrovascular responsiveness (CVR) and cognitive performance. Until now, however, we have not had the opportunity to test the hypothesis that LCn-3PUFA can similarly benefit cognition by improving cerebrovascular function [3]. This paper describes a pilot study to test this hypothesis by supplementing borderline or mildly hypertensive adults with DHA-rich fish oil.

## 2. Methods

### 2.1. Study Design

A 20-week randomised, double-blind, placebo-controlled intervention trial was undertaken at the University of Newcastle’s Clinical Nutrition Research Centre following approval by the Human Research Ethics Committee (H-2014-0152) and registration on the Australian Clinical Trials Register (ACTRN12614000762651). Conducted according to International Conference on Harmonization Guidelines for Good Clinical Practice, the trial was designed to investigate effects of LCn-3PUFA supplementation on cerebral circulatory function, mood and cognitive performance in borderline hypertensive adults, with change in CVR to hypercapnia as the primary outcome. Secondary outcomes included effects of supplementation on clinic blood pressure (BP) and systemic arterial compliance (AC), performance of a battery of neuropsychological tests and the accompanying CVR to these tests, measures of mood states and a range of biomarkers including erythrocyte fatty acid (to derive an Omega-3 Index). The Omega-3 Index (O3I) was used to assess the baseline omega-3 status of participants, their compliance with treatment and correlations between changes in status and changes of both primary and secondary outcomes. We also determined if any changes in CVR to hypercapnia following supplementation were related to other outcome measures.

### 2.2. Participants

We estimated that 48 participants would give 80% power with alpha = 0.05 to detect a 7.5% change in CVR to hypercapnia (relative increase ~25%), based on a 9% SD observed previously [10]. We aimed to recruit a total of 60 participants to allow for 20% attrition. Participants were recruited from the general public via an existing register of volunteers, by distributing flyers and by media advertising. They were selected according to the following criteria:

- Inclusion criteria
  - Aged 40–85 years
  - Clinic SBP between 130–160 mmHg or DBP between 85–100 mmHg (determined at screening)
  - Consuming <2 fish/seafood meals per week
  - Consuming ≤300 mg/day of LCn-3PUFA from fish oil supplements or enriched foods
  - Unlikely to change medication/supplements during the intervention
  - Competent in use of computer mouse and keyboard
- Exclusion criteria
  - Suspected dementia (Modified-Mini Mental (3MS) examination score of <78/100)
  - Smokers or currently on nicotine therapy
  - Neurological conditions
  - Kidney/liver disease/diabetes
  - Major depression as diagnosed by a health care professional
  - Impaired vision
  - Unsatisfactory TCD signal in the middle cerebral artery (MCA)
  - Unwilling to eat or having intolerance to fish or vegetable oil
  - Unwilling to provide a blood sample
  - Unwilling to maintain pre-enrolment physical activity levels and dietary habits during the trial

### 2.3. Clinic Visits

Potential participants were sent detailed information and completed a health and lifestyle questionnaire prior to attending the clinic for further screening to determine study eligibility. They arrived after fasting overnight and their height, weight and waist circumference were measured before assessments of clinic BP and AC. Participants were then fitted with a TCD headpiece which supported ultrasound probes adjacent to each temporal region; probes were adjusted to obtain a suitable blood flow signal in the MCA for the measure of CVR to hypercapnia. Those with BP outside the inclusion range or an unsatisfactory blood flow signal were excluded from the study. The dementia status of the participants was determined using the Australian Version of the 3MS. No volunteers scored below the cut off (78/100; an indicator of suspected dementia). Finally, a blood sample was obtained for erythrocyte fatty acid analysis.

Participants returned to the clinic within 7 days for neuropsychological tests, having fasted for one hour (no food/beverage except water). The TCD headpiece was refitted to participants and probes adjusted to obtain a measurable blood flow signal in the MCA. Continuous recordings of changes in mean blood flow velocities were obtained as they undertook the neuropsychological test battery. Participants were asked to complete a short questionnaire on their LCn-3PUFA intake and a scale to assess current mood states. Their designated supplement (see below) was then dispensed for daily consumption over the next 10 weeks. Participants returned to the clinic after 10 weeks following an 8hr fast (no food or beverages except water) for reassessment of their clinic BP and AC and assessment of CVR to hypercapnia, after which sufficient supplement was dispensed for a further 10 weeks. This visit also served as a check of compliance and the participant’s well-being.

Finally, at the end of the 20-week intervention, participants returned to the clinic with any remaining supplement and the baseline assessment visits were repeated in the same order.

### 2.4. Supplements

Participants were required to consume four capsules of EPAX 1050 fish oil or a corn oil placebo daily for 20 weeks. Each EPAX capsule contained 400 mg DHA and 100 mg eicosapentaenoic acid (EPA), yielding a total dose of 2 g LCn-3PUFA per day. Placebo capsules contained corn oil. Blinding was maintained until all data analysis had been completed. To ensure well-balanced treatment groups, participants were assigned to EPAX or placebo according to Altman’s allocation by minimization method [12] based on their age and BP obtained at the screening visit. The first participant was randomly allocated by a coin toss.

### 2.5. Outcome Assessments

#### 2.5.1. Clinic BP and Arterial Compliance (AC)

After resting quietly in a seated position for 10 min, BP, heart rate and AC readings were taken at 5 min intervals by a single observer using a Cardiovascular Profiler (Cardiovascular Profiler CR2000). An appropriate size blood pressure cuff placed firmly around the brachial artery in the non-dominant arm, while a tonometer was positioned over the dominant radial artery to assess elasticity of large (LAC) and small (SAC) arteries and systemic vascular resistance (SVR) by pulse wave analysis. The first of four consecutive readings was discarded and the remaining measurements were averaged for analysis.

#### 2.5.2. Indices of Cerebrovascular Function

Increases in blood flow velocity in the MCA in response to vasodilator stimuli reflect endothelial dilatation in the distal microvasculature. Basal cerebral blood flow velocity (BFV) measures (peak systolic, end diastolic and mean) and cerebral pulsatility index (PI) were recorded in the MCA over 10 cardiac cycles, after which CVR to hypercapnia was determined. Participants breathed carbogen gas (5% CO_2_, 95% O_2_) for 180 s and the peak increase of BFV was recorded. CVR to hypercapnia (expressed as a percentage) was calculated as follows: 100 × (peak BFV during hypercapnia − basal BFV)/basal BFV.

#### 2.5.3. Neuropsychological Tests

##### Oral Trail Making Task (TMT, a Measure of Executive Function)

Participants are instructed to pair as many number and letter combinations as they could in ascending and alphabetical order (i.e., 1-A-2-B-3-C etc.) in 60 s. One point is given for each correctly identified pair. The maximum points they can achieve is set at 55.

##### Oral Serial Subtractions 3 s (SS3) (Measure of Working Memory)

Participants have to subtract in intervals of 3 from a 3-digit number. They are scored on the number of correct responses made in 60 s. The maximum number of correct responses is set at 90.

##### N-Back Test

Participants view a series of numbers on a screen and are asked to press a button whenever they spot a target number, which is designated as the number that appears one, two or three numbers beforehand. For example, in a 1-back test, the target is any number identical to the number preceding it. The task consists of 6 randomized blocks; each test has 2 blocks lasting 90 s each, with 5 s rest before the next block begins. Correct responses are recorded for each condition, then combined.

##### Computerised Multi-Tasking Test Battery (Purple Framework, UK)

This test battery consists of 4 non-verbal tasks (to minimise potential artefacts from speaking) that assess attention, concentration, processing speed and executive function. Participants are assessed for 2 min on each individual task which is set at a high level of difficulty to evaluate performance. They are then given 5 min to simultaneously complete all 4 tasks set at a moderate level of difficulty, to score their overall multi-tasking performance. The tests appear on a computer screen in the following order:

- Test 1—High number tap (attention): A 4 × 4 grid containing digits between 0 and 9 appears. Participants must highlight the highest digits in each grid by clicking on them (e.g., if the highest digit in the grid is “8”, the participant must click all of the 8 s. Once all of the highest digits are successfully highlighted, the grid refreshes and a new (random) grid appears. Participants complete as many grids as they can within the allocated time.
- Test 2—Letter search (verbal memory): A string of random letters appears in a box for a few seconds. Participants try to remember these letters before they disappear. A single (target) letter then appears in the centre of the screen. If the target letter comes from the original list of letters, they click ‘yes’ on the screen with the mouse, otherwise they click ‘no’. Participants have to select a response within 10 s.
- Test 3—Visual Warning (reaction time): The task starts with six red bars beginning to rise upwards at different speeds. When the highest bar reaches the top, digits appear on each bar and a warning sign flashes on the screen, trying to capture the participant’s attention. Participants respond by clicking on the bars in numerical order (from 1 to 6). Once they click on the bars in correct order, the task resets and a new trial begins automatically.
- Test 4—Stroop colour-word test (executive function): Four coloured blocks (blue, yellow, red and green) appear on the right-hand side of the screen. At a set time interval, a colour name appears to the left of the blocks. Participants click on the coloured block on the right that corresponds with the word, regardless of the colour in which the word is written. For example, if the name “blue” appears in red ink, participants should select the blue coloured block.
- Multi-tasking tests (1 + 2 + 3 + 4): All four tasks appear and run at the same time. Participants attempt the tasks simultaneously and equally to assess their divided attention. To determine accuracy in the multi-tasking test battery, their performance of each component task is compared with their performance when they undertook the task individually. Overall performance on the multi-tasking battery is defined as the average accuracy of performance on the High Number Tap, Letter Search and the Stroop colour-word task. Speed of performance on the multi-tasking test battery is the average response time (msec) for all four-tasks during the 5-min test period. The ratio of accuracy to response time serves as a performance index. A higher index indicates better performance.

##### Dual Tasks with Tapping

Participants are asked to tap a key on the keyboard as quickly as possible with their index finger of their dominant hand whilst performing the oral Trail Making Task, in which they alternate between reciting a number and a letter starting from ‘1’ and ‘A’ and ending with ‘26’ and ‘Z’. They are then asked to tap a key as quickly as possible with their index finger of the non-dominant hand for 60 s whilst performing the Serial Subtractions 3 s task.

##### Go-no-Go Test

This reaction time test is presented on a computer screen where a series of 13 solid-coloured circles or patterned circles appear at random intervals. Participants are asked to press a keyboard space-bar as quickly as possible when a solid-coloured circle appears but ignore the patterned circle.

##### Paper TMT

In Trial A, participants are required to draw a continuous line connecting 25 numbers spread randomly across the page in ascending order. In trial B, they have to draw a continuous line alternating between numbers and letters (1,a,2,b,3,c etc.) spread randomly across the page. The times taken are recorded.

### 2.6. Analysis of the Cognitive Outcomes

Each cognitive test was converted to a z-score and summed to give a measure of global cognitive performance. To quantify treatment changes in performance of the cognitive test battery, the scores obtained for each cognitive test at week 20 were normalised to the cohort’s baseline performance.

### 2.7. CVR to Neuropsychological Tests

Changes in BFV in the MCA during individual cognitive assessments were recorded continuously. CVR to cognitive stimuli was calculated as follows: (100 × peak BFV during a cognitive test − basal BFV obtained prior to the test)/basal BFV. An overall CVR to the neuropsychological test battery was determined by averaging bilateral CVR responses across all tests.

### 2.8. Mood States

After the neuropsychological test battery, participants completed the Profile of Mood States (POMS) questionnaire which consisted of 65-adjectives that they rated on a 5-point Likert scale (1 being “not at all” and 5 being “extremely”) for a variety of different descriptors (e.g., friendly to describe how they had been feeling in the previous week (including the day of the visit) [13].

### 2.9. Erythrocyte Fatty Acid Profiles (Omega-3 Index)

Venous blood samples collected in EDTA tubes were centrifuged (1500 g, 15 min, 4 °C) to separate plasma and erythrocytes. To prevent fatty acid oxidation, butylated hydroxyl toluene was added to erythrocytes prior to storage at −80 °C. Fatty acid profiles of erythrocytes were analysed by gas chromatography following direct transesterification according to Lepage & Roy (ref). Peaks were identified by retention times from calibration curves with reference to fatty acid standards. 

### 2.10. Statistical Analysis

The primary outcome was the effect of LCn-3 PUFA supplementation on CVR to hypercapnia in the MCA. Using SPSS^®^ Version 21 (IBM^®^, New York, United States of America), an ANCOVA with age and 3MS scores as covariates where appropriate was applied to all outcomes to determine the significance of differences between treatment and placebo groups. The level of significance was set at *p* < 0.05. Linear regression analyses were used to test (1) whether treatment changes in CVR of the MCA during hypercapnia as well as during cognitive testing predicted changes in cognitive performance and mood states; (2) relationships between these outcome measures and changes in omega-3 status (erythrocyte LCn-3PUFA levels). All results are presented as mean ± SEM. There is increasing evidence of potential sex differences in responses to LCn-3PUFA treatment [14,15]. Therefore, we also analysed all treatment changes separately for male and female participants.

## 3. Results

### 3.1. Participants

**Figure 1:**
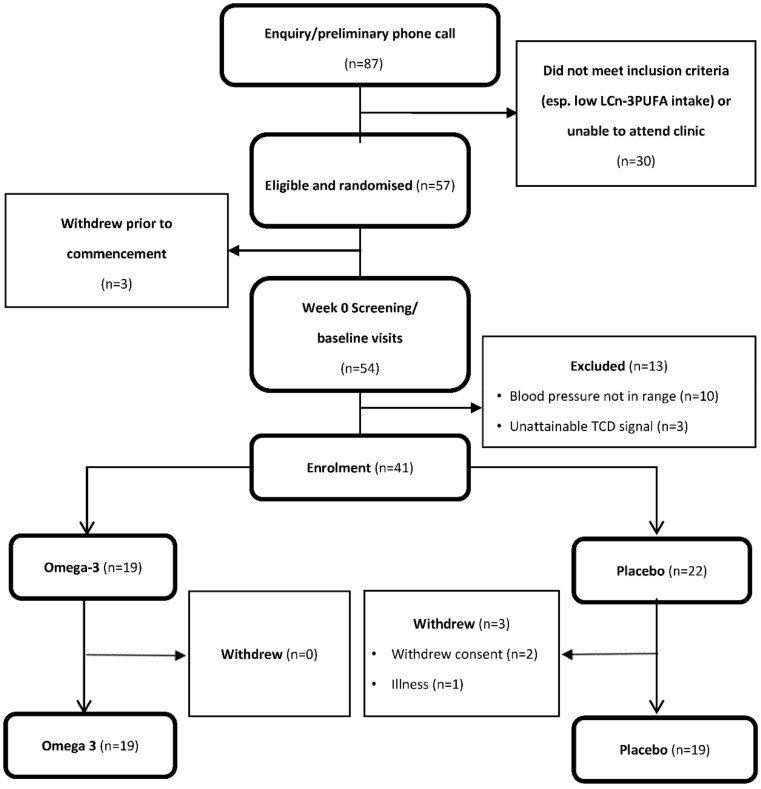
Consort diagram of participants who were screened, enrolled and completed the study.

Figure 1 shows that, of 87 participants who expressed interest in the study, 41 met the criteria for enrolment and 38 (26 males and 12 females) completed the study (the target was 48). Their baseline characteristics appear in Table 1. They averaged 64 years of age, overweight and mildly hypertensive with 15 years of formal education and high 3MS scores indicating normal cognitive function.

Their average seafood consumption (most commonly salmon and tuna) was 125 g per week; only 5 of 38 participants were taking fish oil supplements. Importantly, no participants exceeded the exclusion limit for LCn-3PUFA supplementation (300 mg per day). We estimated the average EPA + DHA intake at baseline to be around 200 mg per day, with no difference between sexes. Compliance with capsule consumption based on capsule counting was similar in both the LCn-3PUFA group and the placebo group (92.6% and 92.0% respectively). Interestingly, 3 of the 19 participants in the placebo group failed to achieve 80% compliance compared with only one individual in the LCn-3PUFA group.

### 3.2. Primary Outcome—CVR to Hypercapnia

**Figure 2:**
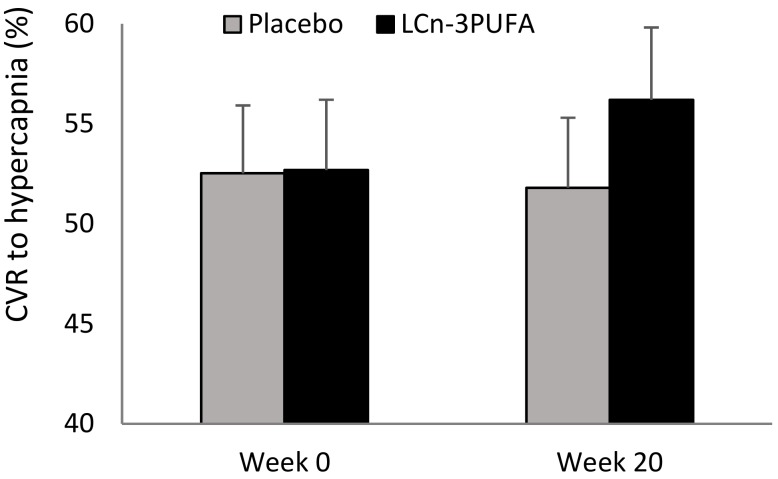
CVR to hypercapnia at baseline and end of intervention in placebo and LCn-3PUFA groups.

Following 20 weeks of supplementation CVR to hypercapnia increased by 3.5 ± 2.1% in the LCn-3PUFA group but decreased by 0.7 ± 2.0% in the placebo group (Figure 2), representing a relative improvement of 8.1%; however, this was not statistically significant (*p* = 0.136). We further examined the influence of sex on the change in CVR and found a significant (*p* = 0.024) improvement of 13.7 ± 4.9% in CVR to hypercapnia following LCn-3PUFA supplementation in women, representing a relative improvement of 26%. The observed difference in men, however, was only 0.5 ± 2.6% and not significant (*p* = 0.860).

### 3.3. Secondary Outcomes

#### 3.3.1. Indices of Systemic and Cerebral Vascular Function

Table 2 shows changes in blood flow haemodynamics for the two treatment groups. No significant improvements were observed in systemic BP or compliance of either large or small arteries; however SVR was significantly reduced following LCn-3PUFA supplementation (*p* = 0.05); this was also attributable to a predominant effect in females. These systemic circulatory benefits were reflected in the cerebrovasculature; PI was significantly reduced by LCn-3PUFA supplementation in all participants (*p* = 0.02) and in women only (*p* = 0.05).

#### 3.3.2. CVR to Cognitive Stimuli (Neurovascular Coupling)

**Figure 3:**
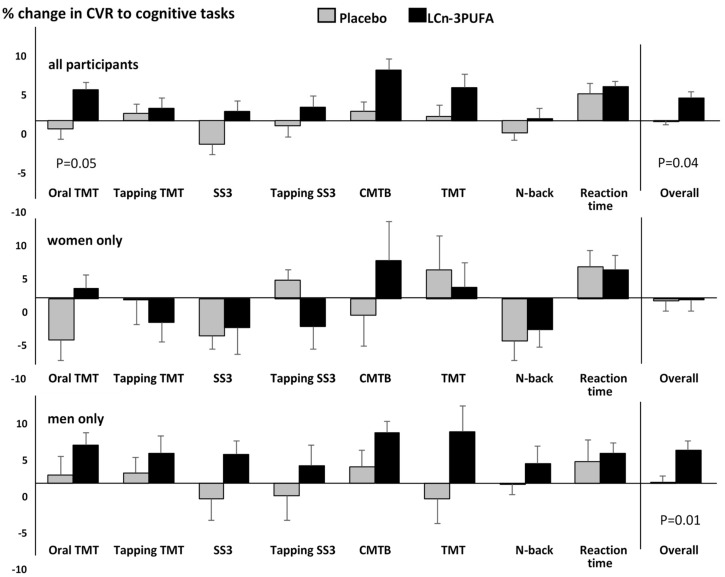
Treatment differences in CVR to the cognitive test battery. TMT—Trail Making Task; SS3—Serial Subtractions 3 s; CMTB—Computerised Multi-tasking Test Battery.

Figure 3 shows changes in CVR during each individual cognitive test as well as the overall CVR to the cognitive test battery for all participants and for men and women only. Supplementation with LCn-3PUFA for 20 weeks significantly improved CVR to the oral TMT test (*p* = 0.05) as well as the overall neurovascular coupling capacity (*p* = 0.04). Moreover, CVRs to the other cognitive tasks tended to be greater after LCn-3PUFA than placebo supplementation, even though the differences did not reach significance. Surprisingly, this tendency appeared to be driven by the responses in men; those taking the LCn-3PUFA supplement exhibited greater improvements in CVR for every cognitive task than those taking the placebo. This is reflected in a significant overall change in CVR to the complete test battery (*p* = 0.01), whereas the overall CVR response in women remained unchanged, despite a significant improvement (*p* = 0.04) in CVR during the oral TMT task.

#### 3.3.3. Cognitive Performance

Table 3 shows the changes in Z-scores for performance of each individual cognitive task as well as the overall cognitive test battery. There were no significant treatment related effects for individual tasks or overall performance in the whole study group or in men and women evaluated separately.

#### 3.3.4. Profile of Mood States

LCn-3PUFA supplementation for 20 weeks did not result in significant improvement of mood states. In fact, the reduction in total mood disturbances appeared more pronounced in the placebo group, although not significant (Table 4). No gender differences were observed, and therefore are not reported.

#### 3.3.5. Erythrocyte Fatty Acid Analysis

Table 5 shows the relative proportions of EPA and DHA (expressed as a percentage of total fatty acids) in erythrocytes and the O3I at baseline and after 20 weeks of supplementation. At baseline, both groups had similar LCn-3PUFA levels and no differences were observed between sexes. Following supplementation for 20 weeks, there were no significant differences in the placebo group. However, LCn-3PUFA supplementation virtually doubled erythrocyte EPA and DHA levels and the O3I (EPA + DHA). The differences between treatment groups were highly significant (*p *< 0.001).

#### 3.3.6. Correlations between Changes in Erythrocyte Fatty Acids and Changes in Outcome Measures

Regression analysis of within-individual responses in all study participants showed that changes in their erythrocyte fatty acid levels (EPA, DHA and O3I) following supplementation were unrelated to changes in CVR to hypercapnia or to changes in CVR to cognitive stimuli except for CVR to the overall cognitive test battery, which correlated significantly with the change in EPA (*r* = 0.381, *p* = 0.032, *n* = 32); this association was even more significant in men (*r* = 0.616, *p* = 0.002, *n* = 23). Interestingly, the change of PI in women was inversely associated with changes in both EPA (*p* = 0.01) and DHA (*p* = 0.04). There were no significant correlations between changes in EPA, DHA or O3I and changes in any measure of mood or cognitive performance. Similarly, changes in BP and AC were unrelated to changes in O3I.

## 4. Discussion

Our pilot study provides supportive evidence that regular consumption of LCn-3PUFA can improve cerebrovascular function. There was a trend toward improvement of our primary outcome measure, viz. CVR to hypercapnia, in the overall study group. This was entirely attributable to a significant 26% improvement in women taking the LCn-3PUFA supplement; there was no hint of a change in men. The observed response to this physiological stimulus reflects a generalised improvement of endothelial vasodilator function in the cerebral microvasculature, consistent with enhanced perfusion of brain regions supplied by the MCA. Importantly, this dynamic change was accompanied by a significant decrease of PI, representing a reduction in the stiffness of cerebral arteries, in women only. These beneficial cerebrovascular effects were accompanied by improvements in systemic measures of arterial function, notably vascular systemic resistance and a non-significant increase in compliance of small (resistance) arteries; these effects were once again manifest in women rather than men.

LCn-3PUFA supplementation for 20 weeks also improved neurovascular coupling. The performance of specific cognitive tasks triggers vasodilation in those brain regions mediating the cognitive processes as their demand for oxygen increases. These localised vasodilator responses generate modest changes of blood flow in the MCA but are nonetheless quantifiable by TCD. CVR to the oral TMT, a measure of executive function, and CVR to the overall battery of cognitive tests were enhanced by LCn-3PUFA supplementation. Surprisingly, however, this was driven by the changes in men; every cognitive test appeared to elicit greater increases of CVR in men supplemented with LCn-3PUFA than in those taking placebo. Moreover, the improvement of CVR to the overall cognitive test battery correlated with increases in erythrocyte levels of EPA, not DHA. This is at odds with our hypothesis that DHA is the primary LCn-3PUFA which enhances cerebral endothelial function, improving blood flow globally and on demand in activated brain regions and thereby improving both mood and cognition [3]. It would appear that the improvements in cerebral perfusion which we have observed following consumption of LCn-3PUFA are more likely to be attributable to increases in EPA than DHA.

While our study was in planning, Jackson et al. [16] published results of a pilot study with a similar objective, viz. to investigate any treatment-related effects of 12 weeks supplementation with DHA- and EPA-rich fish oil on the cerebral haemodynamic response to cognitive tasks. Using near infra-red spectroscopy (NIRS), they obtained evidence that the increase in blood perfusion of the prefrontal cortex which occurred during the tasks was enhanced by the DHA-rich oil but not the EPA-rich oil [17]. Around the same time, Jackson et al. also published results of a larger study which indicated that DHA acted in a dose-dependent manner to improve both neurovascular coupling and measures of sustained attention and reaction time [17]. Neither study included baseline assessments, so the extent of improvement with treatment over time could not be ascertained. Since then, Jackson et al. have published a further study in which they compared changes from baseline in older adults (~60% female) supplemented with DHA-rich fish oil (~1 g DHA/day) or placebo for 6 months [18]. However, this study failed to show any improvement of cerebrovascular function or cognitive performance with DHA supplementation. In another study, Konagai et al. supplemented elderly males with sardine oil and krill oil, both rich in EPA, for 12 weeks and found that, compared to placebo, treatment with both oils improved neurovascular coupling and speed of information processing [19]. However, the benefit was significantly greater with krill oil (attributed to LCn-3PUFA in phospholipid form).

These preceding studies reflect the complexity faced by investigators in designing and interpreting trials to evaluate benefits of LCn-3PUFA on brain function and elucidate the underlying mechanisms, including potential changes in cerebrovascular function. Both Jackson et al. and Konagai et al. used NIRS detection of oxygenated haemoglobin as a surrogate measure to evaluate the capacity of the microvasculature in the cerebral cortex to increase blood flow in response to a cognitive demand, whereas we have used a different approach, viz. TCD quantification of increased BFV in the MCA, to evaluate cerebrovascular dilatation [5]. Our TCD technique can readily detect and quantify vasodilator responses throughout the extensive regions of brain tissue that the MCA supplies but cannot identify locations, whereas NIRS is limited to more superficial cortical regions but is also more localised (region specific) [20]. Thus, with both TCD and NIRS, the magnitude of response will vary according to the location and extent of brain tissue being activated by a specific cognitive task and will differ accordingly between techniques.

The likelihood of detecting an effect of LCn-3PUFA supplementation on cerebrovascular function, mood or cognition will also depend on the daily intake of EPA and/or DHA and the duration of supplementation. Despite the significant increase in overall CVR to the cognitive test battery which we found in men supplemented with LCn-3PUFA in the present study, there was no accompanying change of cognitive performance in either men or women when undertaking any of the individual tests or in the overall summation of their test results. Similarly, there were no significant changes in the various categories of mood or in total mood disturbances following LCn-3PUFA supplementation.

The lack of change in mood or cognition could have been due to inadequate intakes of either EPA or DHA, too brief a period of supplementation or an inappropriate choice of participants for our study. In their earlier trials, Jackson et al. reported dose-related improvements of cerebrovascular function (but not cognition) with DHA rather than EPA [16,17]. Increases of blood flow in the prefrontal cortex during cognitive task performance were enhanced with as little as 450 mg DHA + 90 mg EPA/day [17]. It is surprising, therefore, that their latest study [18] in which they gave twice as much DHA (900 mg/day) for more than twice as long (6 months) failed to replicate their earlier observation. As they suggested, the haemodynamic effect may have diminished over the longer supplementation period. A recent meta-analysis by Ismail indicates that a DHA intake exceeding 500 mg/day is required to improve cognition [21]. We hypothesised that the relatively high dose of DHA that we administered (~2 g/day) would be efficacious, based on the previous 6 month supplementation trial of elderly adults in which this dose of DHA improved both mood and verbal letter fluency [1].

Further considerations include the type of LCn-3PUFA given to participants and their gender and risk profiles. Konagai et al. found that supplementing males aged 61–72 years for 12 weeks with krill oil, delivering 193 mg EPA + 92 mg DHA/day in phospholipid form, improved neurovascular coupling and cognitive performance [19]. It is of interest that the significant enhancements of neurovascular coupling seen in our present study were limited to the male participants of similar age and were strongly correlated with EPA rather than DHA levels in erythrocytes. It appears that EPA may be more efficacious than DHA in men, according to an analysis of platelet-inhibitory effects of LCn-3PUFA supplementation [22], whereas DHA may be more efficacious in women [23]. In our study, however, there was no improvement of cognitive performance in either men or women, possibly because the level of EPA in our DHA-rich oil was too low to influence cognition or simply because, like several other studies, it was an underpowered exploratory study. Another possible contributory factor is the choice of neuropsychological tests, including the computerised multi-tasking test battery. However, we have used similar tests in previous intervention trials of vasoactive nutrient supplementation and shown improvements in task performance as well as neurovascular coupling (1,9,10). In addition, despite having mild hypertension, which is a risk factor for cognitive impairment, the cerebrovascular dysfunction in our participants may not have been sufficiently severe to compromise their cognitive function. Indeed, in an early trial (1) where we reported an improvement of verbal fluency following DHA supplementation, we had preselected an elderly population with mild cognitive impairment. Despite a lack of change in mood or cognition outcomes, the present study adds to a growing number of studies suggesting that LCn-3PUFA supplementation can influence brain functions at least in part by enhancing cerebrovascular function, which may potentially delay future cognitive decline.

One could speculate as to other factors that might influence outcomes, such as the age and current cognitive status of the study participants and the likely extent of endothelial dysfunction in the cerebrovasculature, associated with risk factors such as hypertension or diabetes or the possible influence of inflammatory conditions or menopause. Such factors, together with considerations of type, dose and duration of supplement and potential gender differences in responsiveness should be addressed in the design of future adequately powered intervention trials to fully evaluate the potential for EPA- and DHA-rich oils to counteract microvascular disease in our high-risk aging population and thereby help to prevent premature cognitive decline and dementia.

## Figures and Tables

**Table 1:** Participant’s disposition at baseline.

Characteristics	Placebo (*n* = 19)	LCn-3PUFA (*n* = 19)
Gender (M/F)	13/6	13/6
Age (years)	64.1 ± 2.3	63.2 ± 1.6
3MS score (%)	96.3 ± 0.7	96.8 ± 0.7
BMI (kg/m^2^)	28.8 ± 0.9	26.4 ± 0.8
Waist circumference (cm)	97.9 ± 2.2	93.5 ± 2.9
Clinic systolic BP (mmHg)	141.2 ± 2.0	140.4 ± 1.7
Clinic diastolic BP (mmHg)	79.7 ± 1.7	79.4 ± 1.7
LAC (mL/mmHg × 10)	11.4 ± 0.6	12.7 ± 1.0
SAC (ml/mmHg × 100)	3.3 ± 0.3	3.3 ± 0.4
SVR (dyne·sec·cm^−5^)	1758 ± 56	1755 ± 48
MBFV	46.7 ± 2.9	46.0 ± 1.9
PI	0.86 ± 0.04	0.92 ± 0.05

BMI—Body Mass Index; LAC—large artery compliance; SAC—small artery compliance; SVR—Systemic vascular resistance; MBFV—Mean blood flow velocity; PI—Pulsatility index.

**Table 2:** Treatment differences in measures of arterial function for placebo and LCn-3PUFA groups.

	All Participants			Males			Females		
	Placebo	LCn-3PUFA	*p*	Placebo (*n* = 13)	LCn-3PUFA (*n* = 13)	*p*	Placebo (*n* = 6)	LCn-3PUFA (*n* = 6)	*p*
Systolic BP (mmHg)	−3.1 ± 0.4	−5.0 ± 2.2	0.43	−4.1 ± 0.4	−5.3 ± 2.5	0.90	0.1 ± 0.7	−4.2 ± 2.7	0.52
Diastolic BP (mmHg)	−1.3 ± 1.2	−4.5 ± 1.1	0.12	−2.7 ± 1.8	−8.7 ± 2.2	0.42	6.9 ± 1.6	−4.6 ± 1.4	0.20
LAC (mL/mmHg ×10)	20.1 ± 1.2	18.4 ± 1.1	0.96	25.5 ± 2.1	19.8 ± 1.9	0.73	10.2 ± 0.9	12.1 ± 1.0	0.93
SAC (mL/mmHg ×100)	4.8 ± 1.5	11.0 ± 1.3	0.62	7.9 ± 1.8	10.4 ± 1.7	0.90	−2.1 ± 2.1	14.4 ± 2.5	0.20
SVR * (dyne·s·cm^−5^)	−28.3 ± 25.2	−125.2 ± 22.1	**0.05**	−83.6 ± 30.2	−153.2 ± 33.1	0.4	39.1 ± 35.2	−100.1 ± 32.1	0.08
MBFV	−0.5 ± -2.7	−4.8 ± 2.1	0.27	1.7 ± 2.7	−7.6 ± 4.3	0.07	−4.1 ± 4.4	−2.3 ± 4.6	0.51
PI *	0.02 ± 0.04	−0.03 ± 0.05	**0.02**	−0.04 ± 0.04	−0.00 ± 0.03	0.30	0.23 ± 0.02	−0.09 ± 0.04	**0.05**

See Table 1 for abbreviations. * A more negative value indicates a reduction in resistance/stiffness.

**Table 3:** Z-scores for the cognitive test battery and overall cognitive performance at baseline and end of intervention in placebo and LCn-3PUFA groups; treatment differences are also shown.

	Week 0		Week 20		∆ (Week 20—Week 0)		*p*
	Placebo	LCn-3PUFA	Placebo	LCn-3PUFA	Placebo	LCn-3PUFA	
Oral TMT	−0.1 ± 0.2	0.1 ± 0.2	0.3 ± 0.2	0.3 ± 0.1	0.4 ± 0.9	0.3 ± 0.2	0.65
TMT + tapping	−0.1 ± 0.2	0.1 ± 0.2	0.4 ± 0.2	0.2 ± 0.2	0.5 ± 0.2	0.1 ± 0.2	0.20
Oral SS3	−0.1 ± 0.3	0.1 ± 0.2	0.1 ± 0.1	0.3 ± 0.1	0.2 ± 0.1	0.2 ± 0.1	0.59
SS3 + tapping	0.1 ± 0.3	−0.1 ± 0.2	0.3 ± 0.1	0.1 ± 0.1	0.2 ± 0.1	0.1 ± 0.1	0.53
CMTB	0.2 ± 0.3	−0.2 ± 0.2	0.1 ± 0.2	0.1 ± 0.3	−0.1 ± 0.3	0.2 ± 0.3	0.43
Paper TMT	0.5 ± 0.3	0.2 ± 0.2	−0.1 ± 0.4	0.1 ± 0.3	−0.5 ± 0.4	−0.1 ± 0.4	0.5
N-back	−0.1 ± 0.2	0.1 ± 0.2	0.4 ± 0.2	0.3 ± 0.1	0.5 ± 0.1	0.2 ± 0.1	0.19
Reaction time	0.1 ± 0.3	−0.1 ± 0.2	0.1 ± 0.2	0.3 ± 0.2	0.1 ± 0.2	0.4 ± 0.2	0.35
Overall performance	0.1 ± 0.2	0.1 ± 0.1	0.3 ± 0.1	0.3 ± 0.1	0.2 ± 0.1	0.2 ± 0.1	0.93

**Table 4:** Change in mood states after 20 weeks in the placebo and LCn-3PUFA groups.

Profile of Mood States	Placebo	LCn-3PUFA	Difference (LCn-3 PUFA—Placebo)	*p*-Value
Tension	−2.0 ± 0.7	−1.7 ± 0.7	0.3 ± 0.9	0.78
Depression	−1.4 ± 0.6	0.3 ± 1.0	1.7 ± 1.0	0.15
Anger	−1.3 ± 1.0	0.1 ± 1.0	1.4 ± 1.3	0.33
Fatigue	0.1 ± 1.1	−0.3 ± 0.5	−0.4 ± 0.9	0.97
Confusion	−0.9 ± 0.4	0.0 ± 0.9	0.9 ± 0.9	0.37
Vigour	0.2 ± 1.1	0.6 ± 0.6	0.4 ± 1.0	0.76
Total mood disturbances	−5.9 ± 3.0	−2.1 ± 3.2	3.8 ± 3.9	0.39

**Table 5:** EPA, DHA and O3I (expressed as percentages of total fatty acids) in erythrocytes.

	Placebo			LCn-3PUFA		
	EPA	DHA	O3I	EPA	DHA	O3I
**Baseline**						
All subjects (*n* = 37)	1.38 ± 0.10	4.34 ± 0.23	5.72 ± 0.30	1.24 ± 0.10	4.73 ± 0.23	5.96 ± 0.30
Female (*n* = 11)	1.36 ± 0.23	4.23 ± 0.48	5.58 ± 0.65	1.20 ± 0.23	4.75 ± 0.48	5.95 ± 0.65
Male (*n* = 26)	1.39 ± 0.11	4.39 ± 0.27	5.78 ± 0.34	1.25 ± 0.11	4.71 ± 0.27	5.97±0.34
**Week 20**						
All subjects (*n* = 36)	1.21 ± 0.10	4.24 ± 0.21	5.44 ± 0.25	2.78 ± 0.10	7.96 ± 0.21	10.74 ± 0.25
Female (*n* = 11)	1.10 ± 0.15	4.71 ± 0.34	5.82 ± 0.43	3.07 ± 0.15	8.26 ± 0.34	11.34 ± 0.43
Male (*n* = 25)	1.25 ± 0.12	4.04 ± 0.25	5.29 ± 0.30	2.66 ± 0.12	7.83 ± 0.25	10.49 ± 0.30
**Difference (Week 20—Baseline)**						
All subjects (*n* = 36)	−0.17 ± 0.09	−0.10 ± 0.24	−0.28 ± 0.28	1.54 ± 0.09 *	3.23 ± 0.24 *	4.78 ± 0.28 *
Female (*n* = 11)	−0.26 ± 0.15	0.48 ± 0.32	0.24 ± 0.34	1.87 ± 0.15 *	3.51 ± 0.31 *	5.39 ± 0.34 *
Male (*n* = 25)	−0.14 ± 0.11	−0.35 ± 0.30	−0.49 ± 0.36	1.41 ± 0.11 *	3.1 ± 0.31 *	4.52 ± 0.36 *

EPA—eicosapentaenoic acid; DHA—docosahexaenoic acid; O3I—Omega-3 Index. *significantly different from placebo, *p*<0.001.
