# Correction: Howe et al. “Effects of Long Chain Omega-3 Polyunsaturated Fatty Acids on Brain Function in Mildly Hypertensive Older Adults” *Nutrients* 2018, *10*, 1413

**DOI:** 10.3390/nu11051043

**Published:** 2019-05-10

**Authors:** Peter R. C. Howe, Hamish M. Evans, Julia C. Kuszewski, Rachel H. X. Wong

**Affiliations:** 1 School of Biomedical Sciences and Pharmacy, Clinical Nutrition Research Centre, University of Newcastle, Callaghan, NSW 2308, Australia; hamish.evans@newcastle.edu.au (H.M.E.); Julia.kuszewski@uon.edu.au (J.C.K.); rachel.wong@newcastle.edu.au (R.H.X.W.); 2 Institute for Resilient Regions, University of Southern Queensland, Springfield Central, QLD 4300, Australia

The authors wish to make a correction to the published version of their paper [1], having detected a minor error in Table 2. A corrected version of the table appears below, in which absolute values for the large (LAC) and small (SAC) artery compliance have been replaced with the treatment difference values. We apologize for this change which has no impact on the scientific outcomes or conclusions of the study. The original manuscript will remain online on the article webpage, with reference to this correction.

## Figures and Tables

**Table 2:** Treatment differences in the measures of arterial function for placebo and LCn-3PUFA groups.

	All Participants			Males			Females		
	Placebo	LCn-3PUFA	*p*	Placebo (*n* = 13)	LCn-3PUFA (*n* = 13)	*p*	Placebo (*n* = 6)	LCn-3PUFA (*n* = 6)	*p*
Systolic BP (mmHg)	−3.1 ± 0.4	−5.0 ± 2.2	0.43	−4.1 ± 0.4	−5.3 ± 2.5	0.90	0.1 ± 0.7	−4.2 ± 2.7	0.52
Diastolic BP (mmHg)	−1.3 ± 1.2	−4.5 ± 1.1	0.12	−2.7 ± 1.8	−8.7 ± 2.2	0.42	6.9 ± 1.6	−4.6 ± 1.4	0.20
LAC (mL/mmHg ×10)	2.4 ± 0.9	2.3 ± 0.8	0.96	3.4 ± 1.3	2.8 ± 0.9	0.73	1.0 ± 1.3	1.2 ± 0.8	0.93
SAC (mL/mmHg ×100)	0.2 ± 0.3	0.4 ± 0.3	0.62	0.3 ± 0.5	0.4 ± 0.4	0.90	-0.1 ± 0.1	0.3 ± 0.3	0.20
SVR * (dyne·s·cm^−5^)	−28.3 ± 25.2	−125.2 ± 22.1	**0.05**	−83.6 ± 30.2	−153.2 ± 33.1	0.4	39.1 ± 35.2	−100.1 ± 32.1	0.08
MBFV	−0.5 ± -2.7	−4.8 ± 2.1	0.27	1.7 ± 2.7	−7.6 ± 4.3	0.07	−4.1 ± 4.4	−2.3 ± 4.6	0.51
PI *	0.02 ± 0.04	−0.03 ± 0.05	**0.02**	−0.04 ± 0.04	−0.00 ± 0.03	0.30	0.23 ± 0.02	−0.09 ± 0.04	**0.05**

See Table 1 for abbreviations. * A more negative value indicates a reduction in resistance/stiffness.
